# Assessment of small strain modulus in soil using advanced computational models

**DOI:** 10.1038/s41598-023-50106-3

**Published:** 2023-12-18

**Authors:** Hongfei Fan, Tianzhu Hang, Yujia Song, Ke Liang, Shengdong Zhu, Lifeng Fan

**Affiliations:** 1 Transportation Institute, Inner Mongolia University, Hohhot, 010021 China; 2 Institute of Geotechnical Engineering, Nanjing Tech University, Nanjing, 211816 China; 3 Intelligent Transportation Equipment Inner Mongolia Autonomous Region Engineering Research Center, Hohhot, 010021 China; 4 Department of Civil Engineering, Nanjing University of Aeronautics and Astronautics, Nanjing, 211106 China; 5 Knowledge Management Department, Fujian Yongfu Power Engineering Co., Ltd., Fuzhou, 350000 China

**Keywords:** Civil engineering, Computer science

## Abstract

Small-strain shear modulus ($G_0$) of soils is a crucial dynamic parameter that significantly impacts seismic site response analysis and foundation design. $G_0$ is susceptible to multiple factors, including soil uniformity coefficient ($C_u$), void ratio (*e*), mean particle size ($d_{50}$), and confining stress ($\sigma '$). This study aims to establish a $G_0$ database and suggests three advanced computational models for $G_0$ prediction. Nine performance indicators, including four new indices, are employed to calculate and analyze the model’s performance. The XGBoost model outperforms the other two models, with all three models achieving $R^2$ values exceeding 0.9, RMSE values below 30, MAE values below 25, VAF values surpassing 80%, and ARE values below 50%. Compared to the empirical formula-based traditional prediction models, the model proposed in this study exhibits better performance in IOS, IOA, a20-index, and PI metrics values. The model has higher prediction accuracy and better generalization ability.

## Introduction

The dynamic shear modulus (*G*) of the soil is a critical parameter in seismic site response analysis and dynamic foundation design. Its value decreases as the shear strain amplitude ($\gamma _\text{a}$) increases. When the $\gamma _\text{a}$ is less than $10^{-6}$, *G* is referred to as the small strain shear modulus ($G_0$). Many researchers have conducted studies on $G_0$ for sandy soils using cyclic triaxial tests, bending element tests, and resonant column tests. They have analyzed the influencing factors and proposed predictive models.

Resonant column experiments were employed to investigate the influencing factors on the $G_0$ of pure silica sand. The results indicate that $G_0$ exhibits a power-law growth relationship with increasing the confining stress ($\sigma '$) values. At constant $\sigma '$ values, $G_0$ decreases with the increase of the void ratio (*e*)^1–3^. Similar findings have been observed in the studies conducted by multiple scholars^4–9^.The Hardin model is the most commonly used $G_0$ prediction model^1,3^:

1 $$\begin{aligned} G_0 = Af(e){\left( \frac{\sigma '_0}{P_a}\right) }^n \end{aligned}$$

where ***A*** and n are fitting parameters, ***f(e)*** is the function of *e*, and $P_\text{a}$ is reference stress, usually atmosphere pressure (i.e., 100 kPa).

Particle size distribution is one of the most important factors influencing the $G_0$ of sandy soils. Existing research studies often consider the uniformity coefficient ($C_\text{u}$) and the median particle size ($d_\text{50}$) as characteristic parameters of the particle size distribution when conducting a comprehensive analysis of the effect of particle size distribution on $G_0$.

The impact of particle gradation on the $G_0$ of sandy soils was investigated through resonant column experiments^10^. The findings reveal that $G_0$ increases with the rise of $C_\text{u}$ and $d_{50}$ when relative density ($D_\text{r}$) appear similar, with $d_\text{50}$ exerting more prominent influence compared to $C_\text{u}$. Furthermore, in the Hardin model, the parameter *n* escalates with the increase of $C_\text{u}$. In resonant column frequency sweep experiments conducted on uniformly graded natural sand, when $d_{50}$ is less than 1.8 mm, $G_0$ gradually increases with the enlargement of $d_{50}$^11^. Experimental investigations were conducted using the same methodology on silica sands with varying gradations^12^. The results reveal that when $d_{50}$ remains constant for both *e* and $\sigma '$, its variation does not significantly affect $G_0$. However, an increase in $C_\text{u}$ results in a decrease of $G_0$.Similar results have been observed in resonant column and bending element experiments with uniformly sized glass bead powders of different particle sizes^7^: when *e* remains constant, although $G_0$ decreases with increasing $d_{50}$, this decrease can be considered negligible.

The diversity of soil types and experimental methods has led to variations in predictive models for $G_0$. As a result, predicted results for different types of sandy soils often show significant differences when compared to actual measured data. Machine learning algorithms have the ability to model data based on a large amount of experimental data and adaptively adjust model parameters according to different soil characteristics. This approach allows for a more comprehensive consideration of various influencing factors, ultimately improving the comprehensiveness and accuracy of predictive models. Several researchers have already used machine learning algorithms to build predictive models in this regard.

Probabilistic machine learning has been integrated into predictions of desiccation cracks with uncertain input^13^. Six different machine learning algorithms were analyzed, and it was found that the enhanced XGBT model had greater predictive capacity. Additionally, multivariate robust regression analysis and exponential smoothing time-series analysis were used to address zeolite data, resulting in the development of a novel prediction process that correlates influential variables of zeolite–alkali activated sand^14^.The ANFSI, MLP, and MRM machine learning algorithms were used to develop predictions for $G_0$ and minimum damping ratio ($D_\text{min}$) of sand containing rubber particles^15^. The input parameters for these models are $\sigma '$, fiber content, and rubber content. Neural networks with 3-3-1 and 3-2-1 architectures showed good performance in predicting the damping ratio(*D*) and *G* of mica-sand mixtures^16^. When the Backpropagation Neural Net-work(BPNN) model was used to predict the *G* and *D* of marine clay^17^, the established model showed good predictive performance for marine clay at different strains and depths. In addition, a novel genetic expression programming model was used to predict the normalized shear modulus and damping ratio of sandy soils^18^.

Currently, traditional empirical formulas for predicting $G_0$ are constrained by the limitations imposed by the types of soils in the experimental data and the variety of experimental methods, resulting in limited generalization performance of prediction models. In contrast, machine learning algorithms have demonstrated excellent performance in handling large data sets and predicting soil performance indicators.

In this study, $G_0$ data from several published papers are used to build a G0 database for sandy soils. Three machine learning algorithms-BPNN, Genetic Algorithm-enhanced BP Neural Network (GA-BP), and Extreme Gradient Boosting algorithm (XGBoost) for their strong performance in soil prediction are selected to build prediction models. The input features for these models include soil grading characteristics, such as $C_\text{u}$ and $d_\text{50}$, and state parameters, such as $\sigma '$ and *e*. The goal is to obtain accurate predictions of the $G_0$ for different types of sandy soils. A comparative analysis is performed to evaluate the predictive performance of the three machine learning models for $G_0$ in sandy soils, and a comparison with traditional empirical relationship models is made to validate the effectiveness and accuracy of the machine learning models.

## Data analysis and computational methods

### Data collection and data analysis

In this study, a dataset of 1966 sets of $G_0$ values was obtained from 13 different literatures using various experimental methods such as resonant column tests, bending element tests, and torsional shear tests^6,12,19–29^. The dataset includes a wide range of soil types and particle size distributions, including 481 sets of coral sand, 675 sets of silica sand, and 96 sets of sandy gravel obtained using three different sampling techniques.

According to the above factors affecting $G_0$ of the soil, $C_\text{u}$ and $d_{50}$, which can reflect the grading characteristics, *e*, which reflects the density state, and ${\sigma '}$, which reflects the stress state, are selected as input parameters in this paper. Supplement Table S1 provides essential information about the experiments conducted in each referenced source. Soil classification was performed according to ASTM 2487 standards to achieve uniformity. Specifically, soils with poor particle size distribution and those containing poorly graded gravels were designated SP. Sands with poor grain size distribution and the presence of silt were designated SP-SM. Sands with a well-graded particle size distribution and silt content were labeled SW-SM. Pure silt was classified as ML. Well graded gravels were identified as GW, while poorly graded gravels were identified as GP based on their particle size distribution and characteristics.

**Figure 1:**
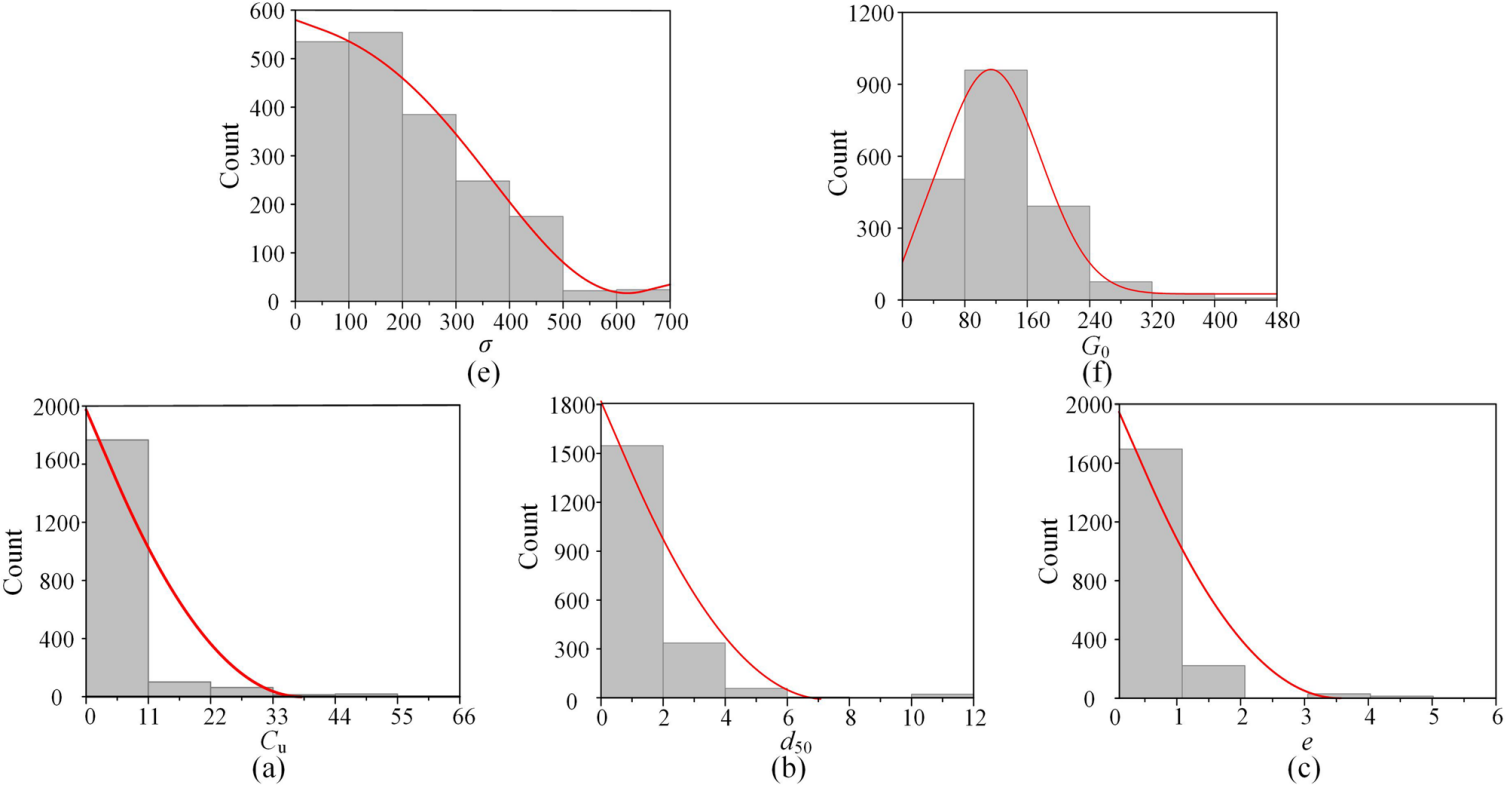
Illustration of normal distribution of parameters: (**a**) $C_\text{u}$, (**b**) $d_{50}$, (**c**) *e*, (**d**) ${\sigma '}$, (**e**) $G_0$.

Each database has numerous data points spread across numerous rows and columns, which makes it challenging to comprehend. Descriptive statistics are thus generated for the database. In the present research, descriptive statistical parameters, mean, SE mean, StDev, variance, coefficient of variance, minimum, Q1, median, Q3, maximum, IQR, skewness and kurtosis have been calculated for overall, training, testing databases, as mentioned in Table 1^30–32^. Table 1 demonstrates that the overall database contains a number of $C_\text{u}$, $d_{50}$, *e*, ${\sigma '}$ and $G_0$ in the range of 1.2–65.49, 0.02–10, 0.25–5.19, 20–700, 12.36–460.79. As a result, Fig. 1 depicts the frequency distribution of the database’s $C_\text{u}$, $d_{50}$, *e*, ${\sigma '}$ and $G_0$ variables. Before utilizing the database for training and testing purposes, the database has been preprocessed, and missing data and outliers have been removed and normalized by the min–max normalization function $=(x$-min)/(max-min), where x is the actual value.

**Table 1:** Results of multicollinearity analysis for the complete database.

values	$C_\text{u}$	$d_{50}$	*e*	${\sigma '}$	$G_0$
Mean	5.17	1.02	0.84	185.55	125.99
SE mean	0.17	0.03	0.01	3.05	1.51
StDev	7.57	1.39	0.56	135.37	67.14
Variance	57.37	1.93	0.31	18325.58	4507.76
Coefficient of variance	1.46	1.36	0.66	0.73	0.53
Minimum	1.2	0.02	0.25	20	12.36
Q1	2	0.2	0.59	98	79.04
Median	2.99	0.55	0.7	150	113.51
Q3	5	1.5	0.92	300	161.33
Maximum	65.49	10	5.19	700	460.79
IQR	3	1.3	0.33	202	82.29
Skewness	4.26	3.73	4.57	1.27	1.21
Kurtosis	21.46	18.38	25.1	1.77	2.31

### Applied soft computing approaches

#### Backpropagation neural network (BPNN) model

**Figure 2:**
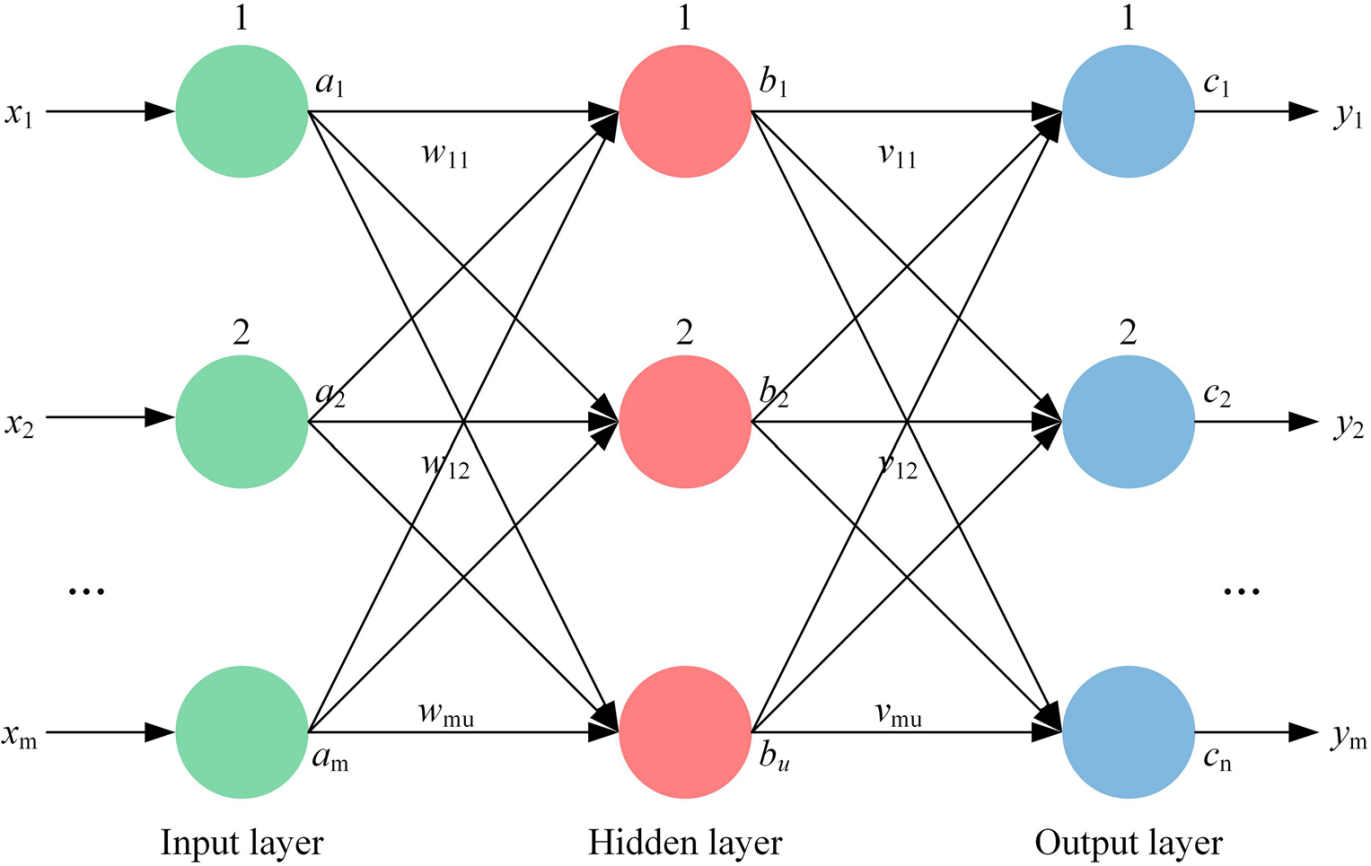
Three-layers BPNN model structure.

Several writers have employed the BPNN modelling approach^33,34^, BPNN is a highly efficient and widely used artificial neural network model that consists of three main layers: the input layer, the hidden layer, and the output layer. Neurons in the input layer can directly interact to receive and process data, while the output layer is responsible for visualizing the results. The neurons in the hidden layer, while not directly visible, play a critical role in processing and transforming the information. Figure 2 shows a schematic of a three-layers BPNN.

The neural network consists of three layers: the input layer represented by $a_\text{m}$, the hidden layer represented by $b_\text{u}$, and the output layer represented by $c$ $_n$. Let $w_\text{mn}$ denote the connection weight between the $m$th neuron in the input layer and the *u*th neuron in the hidden layer, and let $v_\text{mn}$ denote the connection weight between the $u$th neuron in the hidden layer and the nth neuron in the output layer. Thus, we can obtain the expressions for the neurons in the hidden layer and the output layer as given in Eqs. (2) and (3):

2 $$\begin{aligned} b_\text{u}= & {} f\left(\sum _\text{u}{w_\text{mu}}{b_\text{u}}+p_\text{n}\right) \end{aligned}$$

3 $$\begin{aligned} c_\text{n}= & {} f\left(\sum _\text{n}{w_\text{mu}}{b_\text{u}}+p_\text{n}\right) \end{aligned}$$

the excitation function is the Sigmoid function, *k*_**u**_ is the threshold of the hidden layer neuron, *p*_**n**_ is the threshold of the output layer neuron. Each time the output value is compared with the desired output, if the mean square error does not meet the predetermined requirements, the back-propagation process is carried out and the mean square error is returned in the form of a gradient and assigned to each layer neuron. The process is repeated until the mean square error converges.

#### Genetic algorithm-enhanced backpropagation neural network (GA-BP) model

By integrating a genetic algorithm with the Backpropagation Neural Network (BPNN), this approach draws inspiration from natural selection processes found in the biological world. The combination of genetic algorithms with BPNN theoretically ensures that the selection of weights and thresholds becomes an optimisation process. Using the global optimisation advantages of genetic algorithms, the BPNN is extended to improve its global optimisation capabilities. In addition, improvements are made to the genetic algorithm by introducing mechanisms such as “Genetic monitoring” and “Life-death individual alternation”. These enhancements refine the performance of the genetic algorithm.

The “Genetic monitoring” mechanism involves determining the occurrence of genetic degradation by comparing the maximum individual fitness values between the $(n+1)^\text{th}$ and nth generations of a population. Let the maximum individual fitness value of the nth generation be denoted as $Infitmax _\mathrm {(n)}$. If the population fitness satisfies Eq. (4), it is identified as genetic degradation. In such cases, the “Life-death individual alternation” mechanism is activated to increase the diversity of individuals within the population, thereby reducing the probability of genetic degradation. If genetic degradation is not effectively controlled throughout the process, the individual with the highest fitness in the current genetic process is selected as the final outcome.

The formula for calculating individual fitness $Infit_\mathrm {(i)}$ is given in Eq. (5).

4 $$\begin{aligned}{} & {} {Infit_\text{max}}{(n+1)} < {Infit_\text{max}}{(n)} \end{aligned}$$

5 $$\begin{aligned}{} & {} \quad Infit\mathrm {(i)} = 1/{\sum _\mathrm {i=1}^{\text{p}}({y_\text{i}}-{o_\text{i}}})^2 \end{aligned}$$

where **p** is the number of output neurons; *y*_**i**_ is the desired output of the **i**th output neuron; *o*_**i**_ is the network output of the **i**th output neuron.

The “Life-death individual alternation” mechanism involves identifying individuals in the offspring population whose fitness is lower than the average fitness of that population as “deceased individuals”. These “deceased individuals” are then replaced by an equal number of randomly generated “newborn individuals”. The purpose of this mechanism is to preserve the best performing individuals in the current population, while introducing new individuals to prevent the population from becoming less diverse. It helps the algorithm to break out of the current genetic degradation deadlock and prevents it from getting stuck in a “local optimum”. This mechanism is designed to maintain a balance between preserving promising individuals and introducing new genetic material to increase the genetic diversity of the population.

The GA-BP model uses a genetic algorithm during the training process, which uses selection, crossover and mutation operations to perpetuate genetic information. It continuously activates the BP neural network to calculate fitness values for each generation of the population. Throughout this process, the ’Genetic monitoring mechanism’ and the ’Life-death individual alternation mechanism’ come into play until the genetic constraints are met. The individual obtained through genetic evolution is then compared with the individual with the highest fitness value throughout the genetic process. The one with the higher fitness is decoded and assigned to the BP neural network for local optimisation. This process continues until the network’s output meets the required error limits and other criteria, at which point the algorithm is completed.

#### Extreme gradient boosting(XGBoost) model

The Extreme Gradient Boosting algorithm(XGBoost), is an ensemble machine learning algorithm based on decision trees that works within the gradient boosting framework. This algorithm combines several basic learners to create a powerful model. XGBoost is an efficient implementation of the Gradient Boosting Decision Trees (GBDT) algorithm and optimises various aspects of GBDT, including the objective function, the optimisation method, the handling of missing values and the prevention of overfitting^35,36^.

When XGBoost is running, it first trains a basic learner on the training data set. The results produced by this learner are then adjusted based on the training samples, and the next learner is trained using these adjusted samples. This iterative process continues for several rounds until the number of base learners reaches a predefined value. Finally, all the base learners are combined and the computation proceeds as follows:

(1) Constructing Boosting Models:

6 $$\begin{aligned} {f({X_\text{i}})} = \sum _\mathrm {k=1}^{\text{k}}{f_\text{k}}({X_\text{i}}),{f_\text{k}} \in F \end{aligned}$$

where ***F*** is the set of all regression trees.

(2) Training objective function:

7 $$\begin{aligned} {L^{(\text{t})}} = \sum _\mathrm {i=1}^{\text{n}}l({y_\text{i}},{y^{(\mathrm {t+1})}}+{f_\text{t}}({X_\text{i}}))+\Omega {(f_\text{t})} \end{aligned}$$

where “$\sum _\mathrm {i=1}^{\text{n}}l({y_\text{i}},{y^{(\mathrm {t+1})}}+{f_\text{t}}({X_\text{i}}))$” is the loss function and “$\Omega {(f_\text{t})}$” is the sum of all regularisation terms.

(3) The loss function is subjected to a second order Taylor expansion and the final objective function is obtained:

8 $$\begin{aligned} Obj(\text{t}) = \sum _\mathrm {i=1}^{\text{n}}[({\sum _\mathrm {i\in {I_\text{i}}}}g_\text{i}){w_\text{j}}+{\frac{1}{2}}({\sum _\mathrm {i\in {I_\text{i}}}}h_\text{i}+\lambda ){{w_\text{j}}^\text{2}}]+{\gamma }T \end{aligned}$$

### Performance evaluation

The performance of the machine learning models was assessed using several metrics. The mathematical formulation of the performance metrics is as follows^37–39^:

Coefficient of determination ($R^2$)

9 $$\begin{aligned} R^2 = 1 - \frac{{\sum \limits _{\mathrm {i = 1}}^{\text{n}} {{{\left( {{y_\text{i}} - {{{\hat{y}}}_\text{i}}} \right) }^2}} }}{{\sum \limits _{\mathrm {i = 1}}^{\text{n}} {{{\left( {{y_\text{i}} - {{{\bar{y}}}_\text{i}}} \right) }^2}} }} \end{aligned}$$

Absolute relative error(ARE)

10 $$\begin{aligned} \text{ARE}= \left| {\frac{{\left( {{y_\text{i}} - {{{\hat{y}}}_\text{i}}} \right) }}{{{y_\text{i}}}}} \right| \times 100\% \end{aligned}$$

Root Mean Square Error (RMSE)

11 $$\begin{aligned} \text{RMSE }= \sqrt{\frac{1}{\text{n}} \times {{\sum \limits _{\mathrm {i = 1}}^{\text{n}} {\left( {{y_\text{i}} - {{{\hat{y}}}_\text{i}}} \right) } }^2}} \end{aligned}$$

Mean Absolute Error (MAE)

12 $$\begin{aligned} \text{MAE} = \frac{1}{\text{n}} \times \sum \limits _{i = 1}^n {\left| {{y_\text{i}} - {{{\hat{y}}}_\text{i}}} \right| } \end{aligned}$$

Variance Accounted For (VAF)

13 $$\begin{aligned} \text{VAF} = \left[ {1 - \frac{{\text{var}\left( {{y_\text{i}} - {{{\hat{y}}}_\text{i}}} \right) }}{{\text{var}\left( {{y_\text{i}}} \right) }}} \right] \times 100 \end{aligned}$$

a-20 index

14 $$\begin{aligned} \mathrm {a-20 index} = \frac{m20}{H} \end{aligned}$$

Index of Agreement (IOA)

15 $$\begin{aligned} IOA = 1 - \frac{{\sum \limits _{\mathrm {i = 1}}^{\text{n}} {{{\left| {{{\hat{y}}}_\text{i}}-{{y_\text{i}}} \right| }}} }}{2{\sum \limits _{\mathrm {i = 1}}^{\text{n}} {{{\left| {{y_\text{i}} - {{{\bar{y}}}_\text{i}}} \right| }}} }} \end{aligned}$$

Index of Scatter (IOS)

16 $$\begin{aligned} IOS = \frac{RMSE}{Avg.\,of\,Actual\,Values} \end{aligned}$$

Performance Index (PI)

17 $$\begin{aligned} PI = R^2+ (VAF/100)-RMSE \end{aligned}$$

where ${y_\text{i}}$ is the measured value, ${{\bar{y}}}_\text{i}$ is the mean value of $y_\text{i}$, ${{\hat{y}}}_\text{i}$is the predicted value of $y_\text{i}$, *m*20 is the ratio of experimental to the predicted value (0.8–1.2), *H* is the total number of data samples, and **n** is the number of predicted samples.perfect predictive model always has a performance equal to the ideal value mentioned in Table 2.

**Table 2:** Ideal value of the different performance indicators.

Indicators	Value	Indicators	Value	Indicators	Value
$R^2$	1	ARE	0	RMSE	0
MAE	0	VAF(%)	100	a-20 index(%)	100
IOA	1	IOS	0	PI	2

### Sensitivity analysis

**Figure 3:**
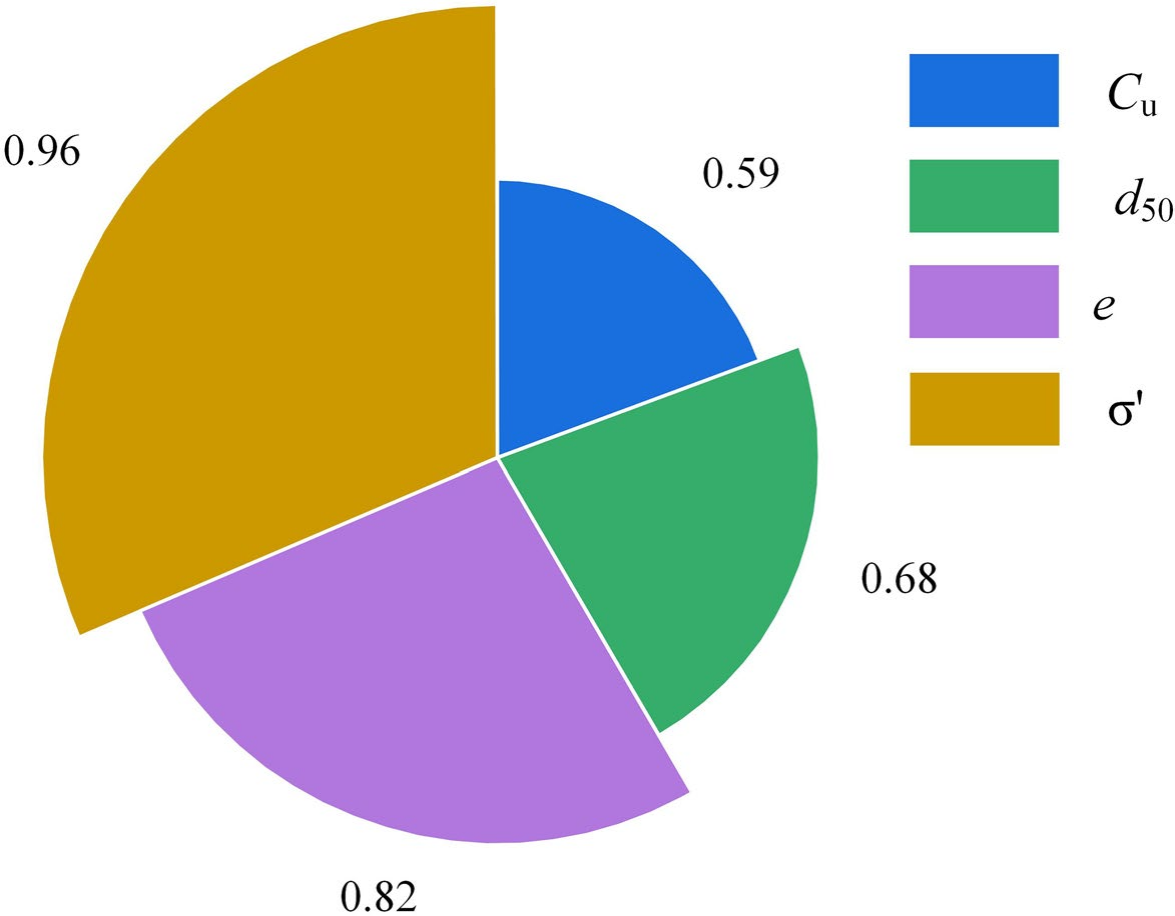
Illustrations of sensitivity analysis for $G_0$.

Sensitivity analysis identifies variables that will most significantly impact predictions. There are global and local forms of sensitivity analysis. Various techniques are used to conduct sensitivity analysis, including the cosine amplitude technique applied in this study. The mathematical expression for the cosine amplitude method is^40,41^:

18 $$\begin{aligned} SS = \frac{{\sum \limits _{c = 1}^n {({X_{ic}}*{X_{jk}})} }}{{\sqrt{\sum \limits _{c = 1}^n {{X_{ic}}^2*\sum \limits _{c = 1}^n {{X_{jk}}^2} } } }} \end{aligned}$$

where $X_{ic}$ is input parameters $C_\text{u}$, $d_{50}$, *e*, and $\sigma '$, and $X_{jk}$ is output parameter $G_0$. A strong influencing input variable always has a *SS* value near one. In this study, 666 data points have been collected from the field. The sensitivity analysis result has been drawn, as depicted in Fig. 3.

Figure 3 illustrates that the input parameters, such as $C_\text{u}$, $d_{50}$, *e*, and $\sigma '$, highly influence $G_0$ prediction. Comparing all input variables, the $C_\text{u}$ variable (= 0.59) influences the $G_0$ prediction less than other input variables.

## Results and discussion

### Modeling

Three methods BPNN,GA-BP and XGB were used to model the prediction of $G_0$.The input parameters for these models included four soil-related factors: $C_\text{u}$, $d_{50}$, *e* and $\sigma '$. The output parameter selected for prediction was $G_0$. Around a third of the data, 666 data points in total, were chosen from the database to be the training set, while the remaining 1300 data points were designated as the test set. The minimum and maximum values and other descriptive statistics of the data in the training set align well with the database and accurately represent the data contained within.

Both the BPNN and GA-BP models used 12 hidden layer neurons, the learning functions are all chosen as trainlm functions, and the transfer functions between the input layer and the implicit layer, and between the implicit layer and the output layer are all chosen as Sigmoid functions.In the case of the XGBoost model, a regression model using XGBRegressor was used. This model was configured with a maximum tree depth of 6, 100 base learners and a learning rate set to 0.1, the rest of the parameters are default values.

**Figure 4:**
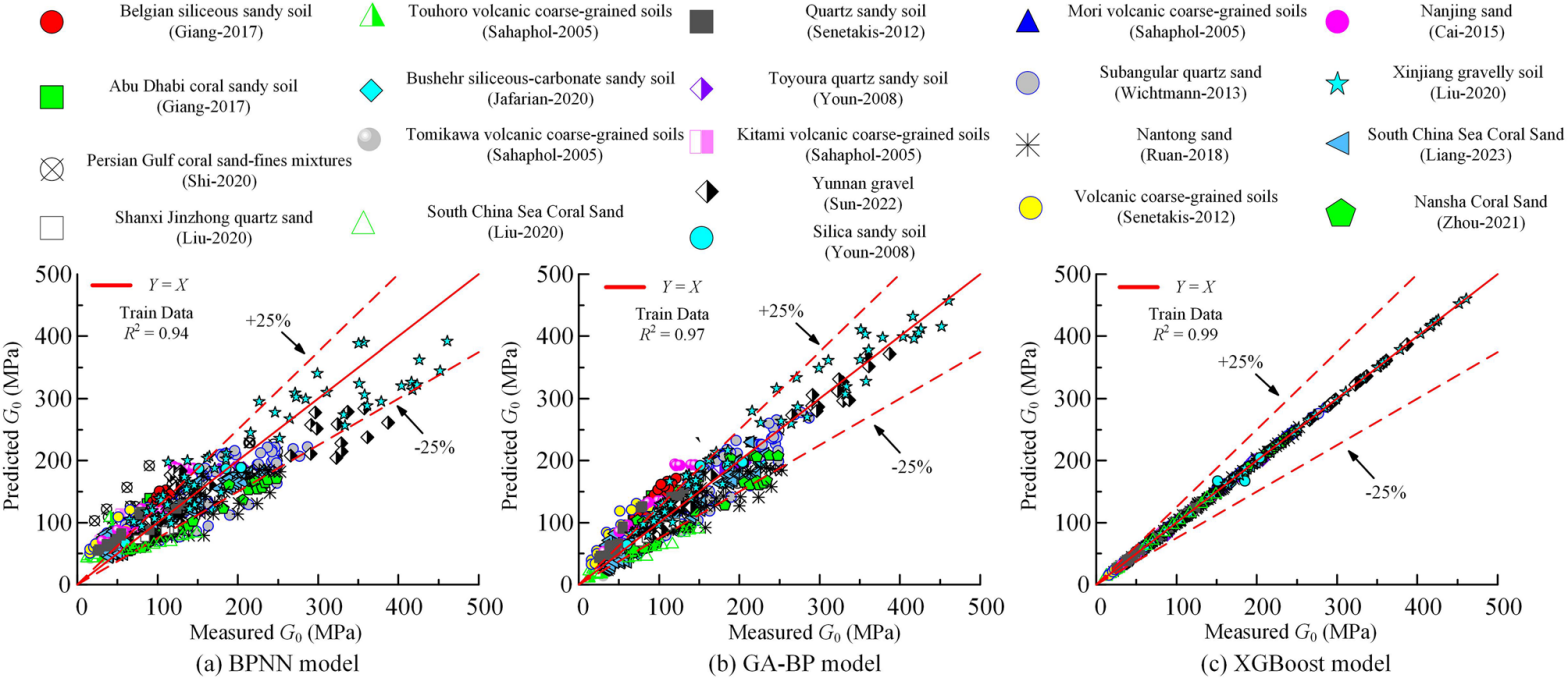
Comparison of the results predicted by different models on the training set with the actual results: (**a**) BPNN model (**b**) GA-BP model (**c**) XGBoost model.

Figure 4 illustrates the comparison between the predicted values of $G_0$ by different prediction models and the actual measured values on the training set. It can be seen that each model can accurately predict the dynamic shear modulus of the soil during training.

Furthermore, the data points for all three models are evenly distributed on both sides of the 45 degree line, indicating reasonably good predictions. Of the models, the XGBoost model shows the best training performance with $R^2$ of 0.99. The ARE is consistently below 10%, with 98% of data points having ARE values below 5%.The GA-BP model comes next with $R^2$ of 0.97 and approximately 67% of the data points have ARE values below 25%. However, the BPNN model has the worst training performance with an $R^2$ of 0.94. Its predictions are less accurate, as only 55% of the data points in the training sets have ARE values below 25%. Consequently, the predictive performance of the BPNN model is comparatively inferior to the other two models. Overall, the XGBoost model shows the highest accuracy in predicting $G_0$ of the training set.

### Model performance analysis

**Figure 5:**
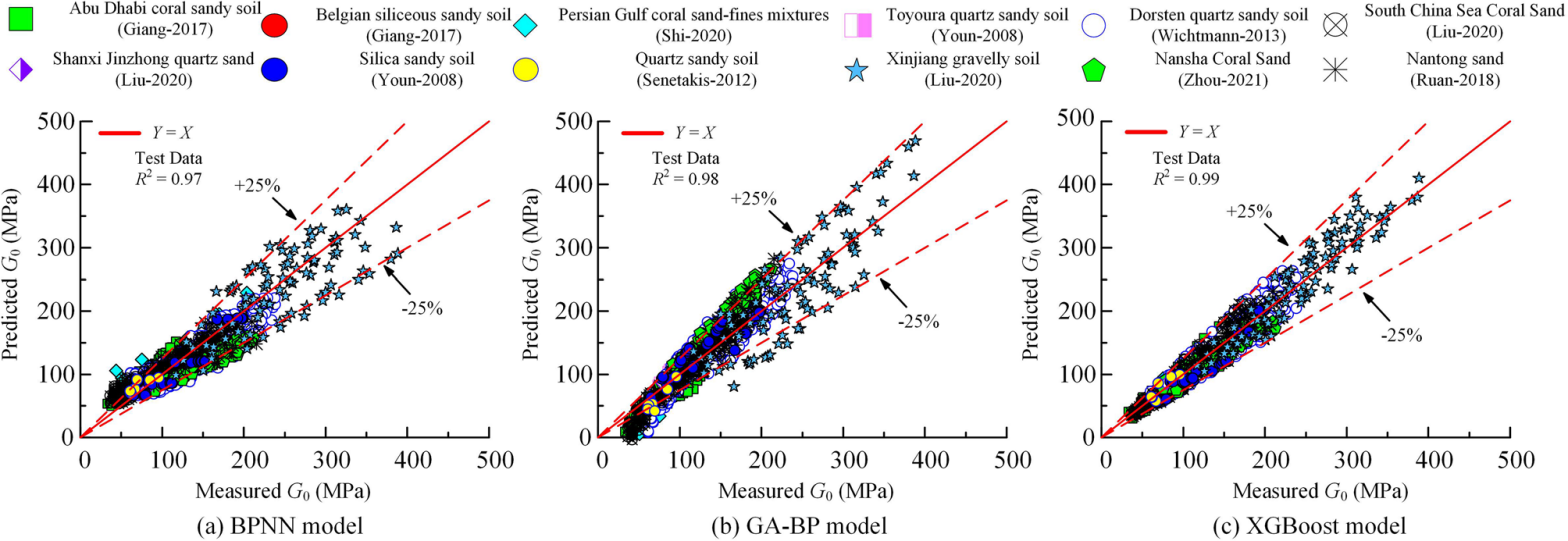
Comparison of the results predicted by different models on the testing dataset with the actual results: (**a**) BPNN model (**b**) GA-BP model (**c**) XGBoost model.

Figure 5 shows a comparison of the predictions made by different prediction models for the $G_0$ on the test data set with the actual measured values. Table 3 shows the performance metrics for the different models. In the figure, the scatter points represent the model predictions for the samples in the test data set. The figure shows that the scatter points for all three models are evenly distributed around the diagonal line (*Y* = *X*), indicating that the models have achieved good predictive performance for $G_0$.

**Table 3:** Different model performance metrics.

Model	Data	$R^2$	RMSE	MAE	VAF/%	IOA	IOS	a-20 index/%	PI
BPNN	Train data	0.94	35.62	28.05	81.56	0.78	0.3	43.09	− 33.87
	Test data	0.97	23.46	18.21	83.77	0.80	0.18	69.00	− 21.65
GA-BP	Train data	0.97	25.57	19.68	90.55	0.84	0.22	56.45	− 23.70
	Test data	0.98	18.75	14.53	89.13	0.84	0.14	83.38	− 16.88
XGBoost	Train data	0.99	1.60	0.97	99.96	0.99	0.01	0.82	0.40
	Test data	0.99	15.54	12.06	92.62	0.86	0.12	90.07	− 13.63

The model with the highest prediction accuracy is the XGBoost model, closely fol-lowed by the GA-BP model, which has slightly lower prediction performance compared to XGBoost. On the other hand, the BPNN model has the worst prediction performance. As can be seen in Fig. 5a, the scatter points of the predictions of the BPNN model are more spread out, indicating less accuracy in its predictions.

**Figure 6:**
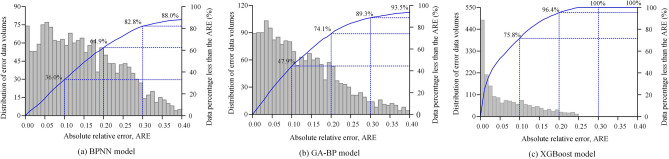
Statistical analysis of predicted and actual ARE values of different forecasting models: (**a**) BPNN model (**b**) GA-BP model (**c**) XGBoost model.

Figure 6 shows the distribution of the ARE for the predictions made by the different models for each dataset in the database compared to the actual measured values. It can be observed that the ARE for all three models is mainly concentrated below 40%.

For the BPNN model, the proportions of data points with ARE less than 10%, 20%, 30% and 40% are 36.0%, 64.9%, 82.8% and 88.0%, respectively. For the GA-BP model, the pro-portions of data points with ARE less than 10%, 20%, 30% and 40% are 47.9%, 74.1%, 89.3% and 93.5%, respectively. The XGBoost model has the highest accuracy, with the propor-tions of data points with ARE less than 10%, 20%, 30% and 40% being 75.8%, 96.4%, 100% and 100% respectively.

The XGBoost model has relatively low ARE values, indicating excellent predictive performance. The GA-BP model slightly outperforms the BPNN model in terms of prediction. As shown in Table 3, the evaluation metrics for the XGBoost model are consistently superior to those of the BPNN and GA-BP models. In summary, these results suggest that the XGBoost model performs best in predicting the $G_0$, while the BPNN model has inferior prediction performance and the GA-BP model also provides good predictions for $G_0$.

### Model performance evaluation

$G_0$ is primarily determined by experiment and empirical equations. Experimental measurements of $G_0$ are subject to many influences, making it difficult to determine its value effectively. Empirical formulae have the advantage of being simple to calculate and easy to use.

In order to evaluate the performance of the computational models, the $G_0$ prediction models proposed by Wichtmann^12^ and Liu et al.^42^ for the calculation of $G_0$ of the soil were selected. Liu et al.^42^ provided an empirical relationship formula for $G_0$ in sandy soils, which takes into account the influence of particle size distribution, based on experimental results of quartz sand and volcanic sand:

19 $$\begin{aligned} G_0 = 108.56{C_\text{u}}^{ - 0.42}\frac{{{{(2.17 - e)}^2}}}{{1 + e}} {\left( \frac{\sigma '_0}{P_\text{a}}\right) } ^{0.36{C_\text{u}}^{0.32}} \end{aligned}$$

Wichtmann and Triantafyllidis based on the results of the silica sand $G_0$ tests, the similar $G_0$ prediction equations are given taking into account the effect of grading:

20 $$\begin{aligned} G_0 = (108.56 + 0.313 {C_\text{u}}^{2.98})\frac{{{{(1.94e^{ -0.066C_\text{u}} - e)}^2}}}{{1 + e}} {\left( \frac{\sigma '_0}{P_a}\right) } ^{0.36{C_\text{u}}^{0.32}} \end{aligned}$$

where $C_\text{u}$ is the uniformity coefficient, *e* is the void ratio, $\sigma '$ is the enclosing pressure, and $P_\text{a}$ is reference stress, usually atmosphere pressure (i.e., 100 kPa).

**Figure 7:**
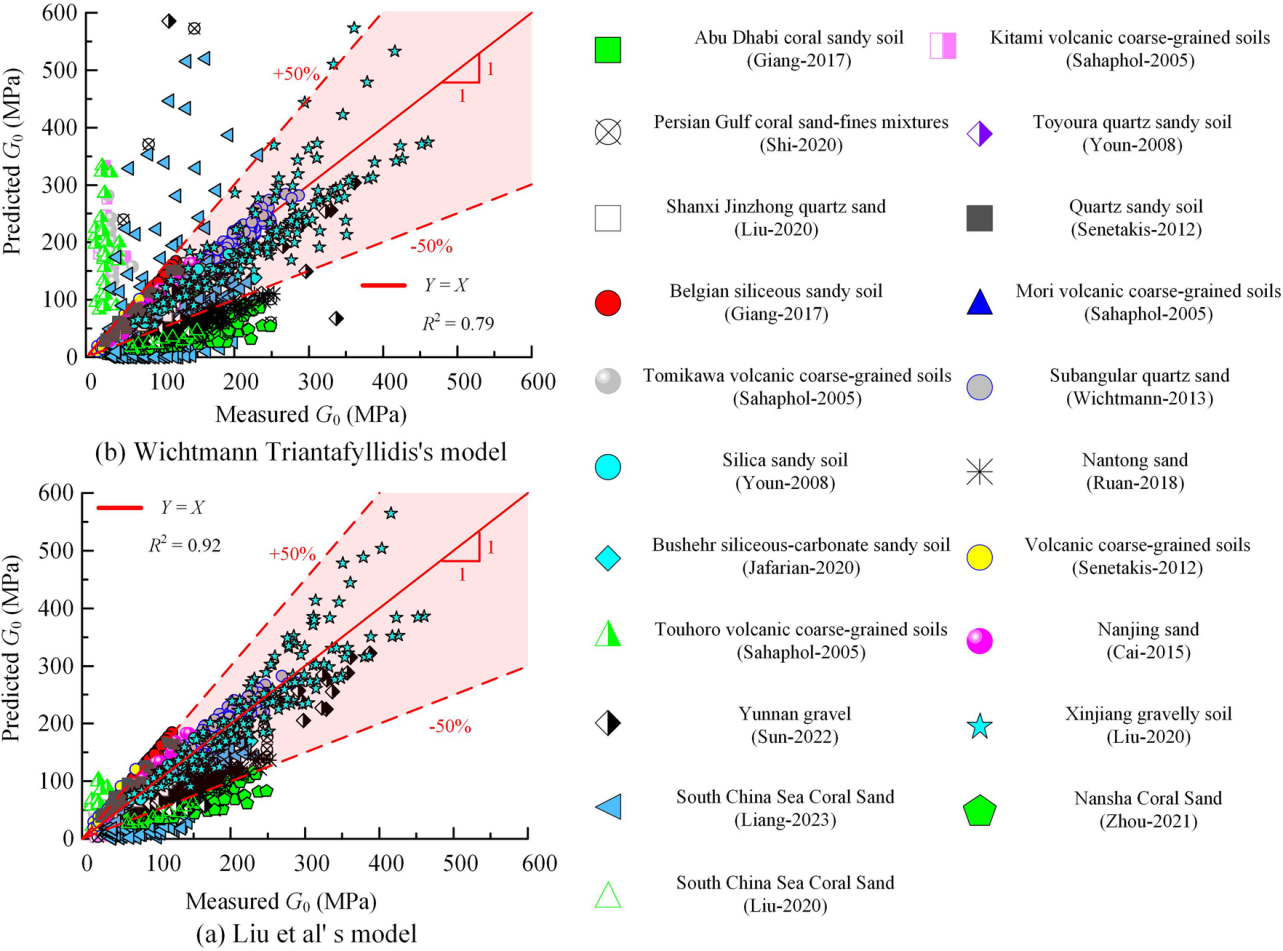
Comparison of predicted and actual values of two traditional empirical formulas: (**a**) Liu et al’ s model (**b**) Witchtmann and Triantafyllidis’s model.

The above two mentioned empirical formulas were used to predict $G_0$ for different types of soils in the database, the comparison between the predicted results and the actual values is shown in Fig. 7. The performance metrics for the traditional empirical formula models and the computational models in predicting the various soil types in the database are summarised in Table 4.

**Table 4:** Performance metrics of predictive models for predicting data in databases.

Model	$R^2$	RMSE	MAE	VAF/%	IOA	IOS	a-20 index/%	PI
BPNN	0.96	28.17	21.55	82.57	0.79	0.22	39.93	− 26.38
GA-BP	0.98	21.31	16.28	89.96	0.84	0.17	48.12	− 19.43
XGBoost	0.99	12.67	8.30	96.47	0.92	0.10	63.53	− 10.71
Liu	0.92	39.19	28.64	72.68	0.72	0.31	30.98	− 67.28
Witchmann	0.79	68.19	42.09	12.41	0.58	0.52	31.62	− 37.54

From Fig. 7. it can be seen that the models proposed by Liu’ s and Wichtmann’s models show significant deviations from the actual values of $G_0$ in the database. Liu ’s model shows that 84.09% of the predicted results have an ARE of less than 50%, while Wichtmann and Triantafyllidis’s model has 63.55% of the results with ARE values of less than 50%. The predicted data are widely scattered, indicating a lower prediction accuracy.

Table 4 shows that the BPNN model, the GA-BP model and the XGBoost model all have R2 greater than 0.95, RMSE less than 30, MAE less than 25 and VAF greater than 80%. In contrast, the traditional empirical formulas provide lower performance metrics across all indicators compared to the three artificial intelligence algorithms, indicating poorer predictive performance.

In summary, the XGBoost model has the highest prediction accuracy for predicting soil $G_0$, followed by the GA-BP model and the BPNN model, while the empirical formula models have the lowest prediction accuracy. Therefore, when performing $G_0$ calculations for soil, it is advisable to select the XGBoost model for estimation.

## Conclusions

The present study has been carried out to introduce exact and dependable computational models for forecasting the $G_0$ of sandy soil. A database of 1966 $G_0$ datasets is established, and 666 of them are extracted as a training set. Three computational models, specifically, BPNN, GA-BP, and XGBoost, are introduced to compare the prediction results with those of the traditional empirical formulas. The research verifies the machine learning model’s superiority for forecasting $G_0$. The study concludes that the utilization of advanced computational models is beneficial for predicting $G_0$. The following conclusions are drawn:

1. Comparing the performance of the three machine learning models, the XGBoost model exhibits the strongest predictive effect for $G_0$, while the GA-BP model, optimized using genetic algorithms, generates more precise prediction results than the BPNN model.
2. The analysis of sensitivity towards four input variables—$C_\text{u}$, $d_{50}$, *e*, and $\sigma '$—using the cosine amplitude method indicated that $\sigma '$ had the most significant impact on $G_0$ prediction. After that, the variable *e* showed a noticeable impact, while $d_{50}$ and $C_\text{u}$ demonstrated lower sensitivities.
3. Comparing the prediction outcomes of machine learning models and traditional empirical formulas, performance metrics such as IOS and IOA demonstrate the former’s superior generalization ability and more accurate prediction performance. Among the machine learning models evaluated, the XGBoost model achieves the best performance in terms of predicting $G_0$.

In summary, this study presents three machine learning models for predicting the $G_0$ of sandy soils. The results show that the XGBoost model performs exceptionally well in predicting $G_0$ in soils, exceeding the predictive power of conventional empirical formulas. However, the machine learning models utilized in this study are limited by the relatively small dataset in the database, resulting in less accurate predictions and restricted prediction accuracy when the models are applied on a larger scale. Hence, it is advisable to integrate Generative Adversarial Network (GAN) algorithms to expand the database, enhance the model’s ability to generalize, and improve predictive accuracy. An efficient and accurate prediction model for $G_0$ is of great significance to engineers working in research areas, including seismic engineering.

### Supplementary Information

Supplementary Table S1.

## Data Availability

All data generated or analyzed during this study are included in this published article and its supplementary information file.
