# Supplementary material for: Assessment of small strain modulus in soil using advanced computational models
Source: Sci Rep. 2023 Dec 18;13:22476. doi: 10.1038/s41598-023-50106-3 (PMC10728178; doi:10.1038/s41598-023-50106-3)
Supplement: Supplementary file 1 — Supplementary Table S1. [file 41598_2023_50106_MOESM1_ESM.docx]

**Assessment of small strain modulus in soil using advanced computational models**

Hongfei Fan^2^, Tianzhu Hang^2^, Yujia Song^1,3^, Ke Liang^4^, Shengdong Zhu^5^, and Lifeng Fan^1,3^,*
^1^Transportation Institute, Inner Mongolia University, Hohhot, 010021, China
^2^Institute of Geotechnical Engineering, Nanjing Tech University, Nanjing, 211816, China
^3^Intelligent Transportation Equipment Inner Mongolia Autonomous Region Engineering Research Center, Hohhot,
010021, China
^4^Department of Civil Engineering, Nanjing University of Aeronautics and Astronautics, Nanjing, 211106, China
^5^Knowledge Management Dept. Fujian Yongfu Power Engineering Co., Ltd., Fuzhou, 350000, China
[*fanlifeng@imu.edu.cn](mailto:*fanlifeng@imu.edu.cn)

This section includes a table for essential information about the experiments conducted in each referenced source (Table S1)

**Table S1** Summary of literature experimental data

| **Literature sources** | **Experiment Type** | **Sandy soil type** | **Methods of sample preparation** | **ID** | **Soil parameters** | | | | | **Soil classification** |
| --- | --- | --- | --- | --- | --- | --- | --- | --- | --- | --- |
|  |  |  |  |  | ***G*_0_** | ***C*_u_** | ***d*_50_** | ***e*_max_** | ***e*_min_** |  |
| Wichtmann andTriantafyllidis(2009) | RC | Subangular quartz sand | AP | L2 | - | 1.5 | 0.2 | 0.994 | 0.595 | SP |
|  |  |  |  | L4 | - | 1.5 | 0.6 | 0.892 | 0.571 | SP |
|  |  |  |  | L6 | - | 1.5 | 2.0 | 0.877 | 0.591 | SP |
|  |  |  |  | L10 | - | 2.0 | 0.6 | 0.865 | 0.542 | SP |
|  |  |  |  | L11 | - | 2.5 | 0.6 | 0.856 | 0.495 | SP |
|  |  |  |  | L12 | - | 3.0 | 0.6 | 0.829 | 0.474 | SP |
|  |  |  |  | L14 | - | 5.0 | 0.6 | 0.748 | 0.395 | SP |
|  |  |  |  | L16 | - | 8.0 | 0.6 | 0.673 | 0.356 | SP |
|  |  |  |  | L17 | - | 2.0 | 2.0 | 0.826 | 0.554 | SP |
|  |  |  |  | L18 | - | 2.5 | 2.0 | 0.810 | 0.513 | SP |
|  |  |  |  | L19 | - | 3.0 | 2.0 | 0.783 | 0.491 | SP |
|  |  |  |  | L21 | - | 5.0 | 2.0 | 0.703 | 0.401 | SP |
|  |  |  |  | L23 | - | 8.0 | 2.0 | 0.520 | 0.398 | SP |
|  |  |  |  | L24 | - | 2.0 | 0.2 | 0.959 | 0.559 | SP |
|  |  |  |  | L25 | - | 2.5 | 0.2 | 0.937 | 0.545 | SP |
|  |  |  |  | L26 | - | 3.0 | 0.2 | 0.920 | 0.541 | SP-SM |
| Giang et al.(2017) | BE | Belgian siliceous sandy soil | MT | Mol | 2.64 | 1.44 | 0.17 | 0.930 | 0.581 | SP |
|  |  | Abu Dhabi coral sandy soil |  | S | 2.79 | 3.46 | 0.73 | 1.330 | 0.903 | SP |
|  |  |  |  | S1 | 2.79 | 1.86 | 0.23 | 1.471 | 0.933 | SP |
|  |  |  |  | VS | 2.79 | 5.43 | 0.43 | 0.956 | 0.508 | SP |
|  |  |  |  | SVS | 2.79 | 5.43 | 0.43 | 1.129 | 0.652 | SP |
|  |  |  |  | SMol | 2.79 | 1.44 | 0.17 | 1.340 | 0.843 | SP |
| Shi et al.(2020) | BE | Persian Gulf coral sand-fines mixtures | DT | S1 | 2.81 | 3.83 | 0.60 | 1.188 | 0.728 | SP |
|  |  |  |  | S1+10%FC | 2.81 | 10.53 | 0.55 | 1.127 | 0.604 | SW-SM |
|  |  |  |  | S1+20%FC | 2.80 | 27.56 | 3.59 | 0.988 | 0.516 | SW-SM |
|  |  |  |  | S1+30%FC | 2.80 | 42.66 | 0.38 | 0.946 | 0.498 | SW-SM |
|  |  |  |  | S1+40%FC | 2.79 | 50.96 | 0.27 | 1.051 | 0.517 | SW-SM |
|  |  |  |  | S1+50%FC | 2.79 | 52.64 | 0.06 | 1.067 | 0.576 | SW-SM |
|  |  |  |  | 100%FC | 2.76 | 65.49 | 0.02 | 1.649 | 0.993 | ML |
| Youn et al.(2008) | RC,BE,TS | Toyoura quartz sandy soil | AP | - | 2.65 | 1.29 | 0.20 | 0.982 | 0.617 | SP |
|  |  | Silica sandy soil |  | - | 2.63 | 2.01 | 0.16 | 0.854 | 0.642 | SP |
| Liu et al.(2020) | RC | South China Sea Coral Sand | DT | 1,2,3,4,5,6,7,8,9 | 2.84 | 2.21 | 0.56 | 1.231 | 0.812 | SP |
|  |  | Shanxi Jinzhong quartz sand |  | 10,11,12,13,14,15,16,17,18 | 2.64 | 2.21 | 0.56 | 0.961 | 0.557 | SP |
| Zhou(2020) | RC | Nansha Coral Sand | DT | - | 2.76 | 1.20 | 0.375 | 1.42 | 0.953291 | SP |
|  |  |  |  | - | 2.76 | 1.20 | 0.750 | 1.260 | 0.902 | SP |
|  |  |  |  | - | 2.76 | 1.20 | 1.5 | 1.270 | 0.969 | SP |
|  |  |  |  | - | 2.76 | 1.20 | 2.5 | 1.390 | 1.085 | SP |
|  |  |  |  | - | 2.76 | 1.8 | 0.375 | 1.349 | 0.869 | SP |
|  |  |  |  | - | 2.76 | 1.8 | 0.750 | 1.279 | 0.855 | SP |
|  |  |  |  | - | 2.76 | 1.8 | 1.5 | 1.140 | 0.896 | SP |
|  |  |  |  | - | 2.76 | 1.8 | 2.5 | 1.296 | 1.006 | SP |
|  |  |  |  | - | 2.76 | 2.4 | 0.375 | 1.163 | 0.755 | SP |
|  |  |  |  | - | 2.76 | 2.4 | 0.750 | 1.213 | 0.774 | SP |
|  |  |  |  | - | 2.76 | 2.4 | 1.5 | 1.067 | 0.835 | SP |
|  |  |  |  | - | 2.76 | 2.4 | 2.5 | 1.233 | 0.970 | SP |
| Senetakis et al.(2012) | RC | Quartz sandy soil | DT | N1 | 2.67 | 1.58 | 0.27 | 1.008 | 0.608 | SP |
|  |  |  |  | N2 | 2.67 | 2.76 | 0.56 | 0.841 | 0.467 | SP |
|  |  |  |  | N3 | 2.67 | 1.34 | 0.60 | 0.963 | 0.628 | SP |
|  |  | volcanic coarse-grained soils | - | - | - | - | - | - | - | - |
| Liu et al.(2021) | BE | Xinjiang gravelly soil | AP | A-2 | 2.63 | 1.45 | 1.5 | - | - | GP |
|  |  |  |  | A-5 | 2.63 | 2.80 | 1.50 | - | - | GP |
|  |  |  |  | A-10 | 2.63 | 5.33 | 0.75 | - |  | GW |
|  |  |  |  | A-20 | 2.63 | 12.90 | 0.55 | - | - | GW |
|  |  |  |  | A-40 | 2.63 | 20.15 | 0.46 | - | - | GW |
|  |  |  |  | B-40 | 2.63 | 4.44 | 0.77 | - | - | GW |
|  |  |  |  | B-20 | 2.63 | 4.64 | 0.86 | - | - | GW |
|  |  |  |  | B-10 | 2.63 | 4.81 | 0.78 | - | - | GW |
|  |  |  |  | B-5 | 2.63 | 4.56 | 0.82 | - | - | GW |
| Liang et al（2023） | RC | South China Sea Coral Sand | DT | N-CU1 | 2.77 | 2.10 | 0.55 | 1.360 | 0.751 | SP |
|  |  |  |  | N-CU2 | 2.77 | 2.47 | 0.53 | 1.182 | 0.646 | SP |
|  |  |  |  | N-S0 | 2.77 | 3.35 | 0.52 | 1.162 | 0.631 | SP |
|  |  |  |  | N-CU3 | 2.77 | 5.99 | 0.52 | 0.958 | 0.516 | SP |
|  |  |  |  | N-CU4 | 2.77 | 11.20 | 0.52 | 0.890 | 0.453 | SP |
|  |  |  |  | N-D1 | 2.77 | 3.05 | 0.21 | 1.165 | 0.582 | SP |
|  |  |  |  | N-D2 | 2.77 | 3.35 | 1.05 | 1.162 | 0.646 | SP |
|  |  |  |  | N-D3 | 2.77 | 2.99 | 1.45 | 1.148 | 0.650 | SP |
|  |  |  |  | N-D4 | 2.77 | 3.26 | 2.00 | 1.183 | 0.733 | SP |
|  |  |  |  | N-FC1 | 2.77 | 4.40 | 0.49 | 1.162 | 0.631 | SP |
|  |  |  |  | N-FC2 | 2.77 | 8.03 | 0.46 | 1.019 | 0.450 | SW-SM |
|  |  |  |  | N-FC3 | 2.77 | 24.46 | 0.43 | 1.009 | 0.412 | SM |
|  |  |  |  | N-FC4 | 2.77 | 29.25 | 0.40 | 0.999 | 0.375 | SM |
|  |  |  |  | N-FC5 | 2.77 | 32.29 | 0.34 | 0.958 | 0.369 | SM |
|  |  |  |  | N-FC6 | 2.77 | 30.22 | 0.27 | 1.025 | 0.401 | SM |
|  |  | Xisha Coral Sand |  | X-CU1 | 2.81 | 2.10 | 0.55 | 2.132 | 1.455 | SP |
|  |  |  |  | X-CU2 | 2.81 | 2.47 | 0.53 | 1.999 | 1.328 | SP |
|  |  |  |  | X-CU3 | 2.81 | 11.20 | 0.52 | 1.430 | 1.050 | SP |
| Jafarian and Javdanian(2020) | RC | Bushehr siliceous-carbonate sandy soil | AP | 1,4,7,10,13,16 | 2.70 | 3.43 | 0.35 | 0.882 | 0.671 | SP |
| Sahaphol and Miura(2015) | BE | Mori volcanic coarse-grained soils | AP | - | 2.82 | 2.4 | 0.6 | 1.257806 | 0.774701 | SP |
|  |  | Kitami volcanic coarse-grained soils |  | - | 2.47 | - | 0.32 | 1.845622 | 1.043011 | SP |
|  |  | Tomikawa volcanic coarse-grained soils |  | - | 2.22 | 4.00 | 1.4 | 3.774194 | 2.950178 | SP |
|  |  | Touhoro volcanic coarse-grained soils |  | - | 2.55 | 3.3 | 5.1 | 3.450262 | 2.507565 | GP |
| Cai et al.(2015) | BE | Nanjing sand | DT | NJ-0 | 2.66 | 1.771 | 0.173 | 1.142 | 0.625 | SP |
|  |  |  |  | NJ-5 | 2.66 | 1.827 | 0.168 | 1.138 | 0.584 | SP |
|  |  |  |  | NJ-10 | 2.66 | 2.467 | 0.163 | 1.131 | 0.535 | SP-SM |
|  |  |  |  | NJ-14.5 | 2.66 | 3.750 | 0.158 | 1.124 | 0.508 | SP-SM |
|  |  |  |  | NJ-20 | 2.67 | 6.000 | 0.152 | 1.123 | 0.469 | SM |
| Sun et al.(2022) | BE | Yunnan gravel | DT | MG-1,2,3 | 2.640 | 13.329 | 0.415 | - | - | SW |
|  |  |  |  | MG-4,5,6 | 2.639 | 14.407 | 0.493 | - | - | SW |
|  |  |  |  | MG-7,8,9 | 2.638 | 15.879 | 0.631 | - | - | SW |
|  |  |  |  | MG-10,11,12 | 2.636 | 35.171 | 1.268 | - | - | SW |
|  |  |  |  | MG-13,14,15 | 2.636 | 47.554 | 2.595 | - | - | SW |
|  |  |  |  | MG-16,17,18 | 2.635 | 46.052 | 5.000 | - | - | SW |
|  |  |  |  | MG-19,20,21 | 2.634 | 39.036 | 5.612 | - | - | GW |
|  |  |  |  | MG-22,23,24 | 2.633 | 27.139 | 6.095 | - | - | GP |
|  |  |  |  | MG-25,26,27 | 2.632 | 9.833 | 6.652 | - | - | GP |
|  |  |  |  | NG-1,2,3 | 2.64 | 11.188 | 0.589 | - | - | SW |
|  |  |  |  | NG-4,5,6 | 2.67 | 4.282 | 0.615 | - | - | SW |
|  |  |  |  | NG-7,8,9 | 2.64 | 6.811 | 0.685 | - | - | SW |
|  |  |  |  | NG-10,11,12 | 2.64 | 18.505 | 1.428 | - | - | SW |
|  |  |  |  | NG-13,14,15 | 2.663 | 25.256 | 6.138 | - | - | GP |
